# Tiered Neuroscience and Mental Health Professional Development in Liberia Improves Teacher Self-Efficacy, Self-Responsibility, and Motivation

**DOI:** 10.3389/fnhum.2021.664730

**Published:** 2021-05-11

**Authors:** Kara Brick, Janice L. Cooper, Leona Mason, Sangay Faeflen, Josiah Monmia, Janet M. Dubinsky

**Affiliations:** ^1^The Carter Center Mental Health Program, Monrovia, Liberia; ^2^Peace Corps Liberia, Monrovia, Liberia; ^3^Ministry of Education, Monrovia, Liberia; ^4^Department of Neuroscience, University of Minnesota, Minneapolis, MN, United States

**Keywords:** neuroeducation, teacher professional development, teacher self-efficacy, teacher self-responsibility, teacher motivation, affective and motivational attitudes, teacher competencies, mental health literacy

## Abstract

After acquiring knowledge of the neuroscience of learning, memory, stress and emotions, teachers incorporate more cognitive engagement and student-centered practices into their lessons. However, the role understanding neuroscience plays in teachers own affective and motivational competencies has not yet been investigated. The goal of this study was to investigate how learning neuroscience effected teachers’ self-efficacy, beliefs in their ability to teach effectively, self-responsibility and other components of teacher motivation. A pilot training-of-trainers program was designed and delivered in Liberia combining basic neuroscience with information on social, emotional, behavioral and mental health issues faced by students. Tier I of the professional development was a 2 weeks workshop led by a visiting neuroscientist. A subset of the 24 Tier I secondary science teachers formed a Leadership Team who adapted the content to the Liberian context and subsequently led additional workshops and follow-up sessions for the Tier II secondary science teachers. Science teachers in both tiers completed the affective-motivational scales from the internationally vetted, multiscale Innovative Teaching for Effective Learning Teacher Knowledge Survey from the OECD. Tier II teachers completed the survey in a pre-post-delayed post design. Tier I teachers completed the survey after the workshop with their attitudes at that time and separately with retrospective projections of their pre-workshop attitudes. Ten of the 92 Tier II teachers participated in structured interviews at follow-up. Statistical analysis of survey data demonstrated improved teacher self-efficacy, self-responsibility for student outcomes, and motivation to teach. Qualitatively, teachers expressed more confidence in their ability to motivate students, engage them through active learning, and manage the class through positive rather than negative reinforcement. Teachers’ own self-regulation improved as they made efforts to build supporting relationships with students. Together, these results demonstrated that (i) teacher affective-motivational attitudes can be altered with professional development, (ii) basic neuroscience, as knowledge of how students learn, can improve teacher competency, and (iii) a training-of-trainers model can be effective in a low and middle income country for disseminating neuroscience knowledge, increasing teachers’ knowledge of students’ social and emotional needs, and promoting educational improvement.

## Introduction

Seminally, Shulman (1987) divided the knowledge base needed by teachers into seven categories: content knowledge, general pedagogical knowledge, pedagogical content knowledge, curricular knowledge, knowledge of learners and their characteristics, knowledge of educational systems and contexts, and knowledge of educational theories and philosophy. Regarding knowledge of learners, Shulman (1986) states “aspects of physiology are apparently deemed necessary because of the expectation that teachers understand the biological functioning of their pupils.” Neuroscience provides teachers with knowledge about how learning occurs in the brain, a topic that falls within the knowledge of students category. More recently, neuroscience was recognized as providing fundamental contributions to knowledge of the learning processes and knowledge of individual student characteristics (Voss et al., 2011). Knowledge of learning processes encompasses understanding memory and information processing, cognitive development, motivation and attention—elements of contemporary neuroscience (Posner and Rothbart, 2007; Voss et al., 2011). Knowledge of individual student characteristics includes specific learning disabilities, such as ADHD, with defined neurobiological bases (MTA Cooperative Group, 1999; Voss et al., 2011). Neuroscience provides a biological basis for how learning occurs in the brain and as such should function to inform theories of learning (Meltzoff et al., 2009; Ansari et al., 2017). Indeed, calls have been made to make neuroscience a part of pre-service teacher education and in-service professional development as part of teachers’ knowledge of students, where it compliments theories of learning from cognitive science and psychology (Dubinsky et al., 2013; Ansari et al., 2017).

Professional development in neuroscience has been demonstrated to increase teacher content knowledge as well as confidence in and use of student-centered pedagogy (MacNabb et al., 2006b; Roehrig et al., 2012). This was true for science teachers and non-science teachers (Schwartz et al., 2019). After attending a series of neuroscience seminars, teachers embraced the content as relevant to their practices (Dommett et al., 2011). Even a short 90 min exposure to neuroscience ideas may produce changes in teachers’ intended classroom practices (Howard-Jones et al., 2020). Prior data (Dubinsky et al., 2019) suggest teachers’ may also provide students with more social and emotional support following neuroscience professional development (PD), but this has not been studied explicitly. Thus, knowledge of how learning occurs in the brain has the power to influence teachers’ thinking about their students and practices. However, the effect of neuroscience training upon established measures of teacher competence have not been previously reported.

Teacher’s competence has been described to “comprise all the required cognitive knowledge for creating effective teaching and learning environments” (Guerriero, 2017b). The international Innovative Teaching for Effective Learning (ITEL) project was designed to assess in-service and pre-service teachers’ professional qualities (Baumert and Kunter, 2013; Blomeke, 2017; Sonmark et al., 2017). ITEL expanded upon Shulman’s (1986, 1987) ideas and conceptualized teachers’ competence as falling into the three broad areas; pedagogical knowledge, opportunities to learn, and affective-motivational attitudes (Guerriero and Revai, 2017; Sonmark et al., 2017). These broader competencies incorporated ideas that go beyond pedagogy and content to address teacher attitudes toward their own learning, motivations and aptitudes (Blomeke, 2017). Teacher competencies and classroom actions are both cognitive, knowledge based and affective, intuitive, and goal driven (affective-motivational) (Blomeke, 2017; Sonmark et al., 2017). The context-dependent choices teachers make are based upon their beliefs about both teaching and their own abilities (Sonmark et al., 2017). Teachers’ own emotional or affective states become central to the process of encouraging and optimizing student learning (Sonmark et al., 2017). Teachers’ social and emotional behaviors set the tone for classrooms. Poor social-emotional skills can exacerbate poor student outcomes while teachers who are competent in these areas can effectively promote social, emotional and intellectual learning among their students (Osher et al., 2012). The ITEL project teacher knowledge survey (ITEL-TKS), based upon these competency dimensions, incorporated many previously developed constructs measuring teacher characteristics (Sonmark et al., 2017). ITEL-TKS operationalized the affective-motivational dimension with scales exploring teacher self-efficacy (TSE), self-responsibility, personal motivation and commitment to teach, goals, and beliefs about instruction and classroom management (Sonmark et al., 2017). As an internationally validated instrument, the ITEL-TKS framework and affective-motivational scales were selected for use in the current study of the effect of neuroscience PD.

The influence of neuroscience on a range of teachers’ attitudes was studied as part of a training program combining neuroscience and mental health (MH) education to secondary science teachers in Liberia. The program’s intent was to improve teachers’ ability to recognize and support students with social, emotional, behavioral or mental health issues. We reasoned that the MH content would be best understood after building a foundational knowledge of how the brain worked. The workshop had two main goals. The first was to influence teachers’ attitudes toward persons with MH concerns by providing the background needed to understand student MH issues and to provide referrals to local MH clinicians (Weist et al., 2017). The effects of the combined training on teachers’ attitudes toward mental illness (MI) appear elsewhere (Brick et al., 2021). The second goal was to promote teacher beliefs about their abilities to motivate, engage and involve students in active learning, as aligned with the goals for Education for Sustainable Development (Ahmad et al., 2018; Education 2030, 2018). The latter goal required providing teachers with knowledge about the workings of the brain, a subset of the broader category of knowledge of students. This paper focuses on how the combined neuroscience and MH training may have altered teacher affective and motivational attitudes toward their perceived ability to reach and engage students.

To that end, a professional development program was designed and piloted to provide Liberian secondary science teachers with an intellectual foundation for understanding student social, emotional and behavioral needs combined with modeling of inclusive pedagogical practices. The content focus was neuroscience, content included in the Liberian secondary school curriculum that would also advance interest in STEM, a stated economic need for African development (African Development and Bank, 2020). On a theoretical level, neuroscience and MH were viewed as part of knowledge of students (Shulman, 1987). The MH portion of the intervention was designed to promote teacher recognition of and response to social, emotional and behavioral student issues (Pacione and Cooper, 2014). In this frame, the workshops were designed to influence teachers’ professional competence in the affective-motivational aspects of their overall teaching (Guerriero, 2017a).

Understanding both the neuroscience of learning and memory and students’ social, emotional and behavioral issues comprise different aspects of knowledge of students and their characteristics. As such, this information would be expected to influence teachers’ self-efficacy (TSE), their attitudes and beliefs around instructional strategies, classroom management, and student engagement. Viewing TSE within the larger dimension of teacher affective-motivational attitudes (Sonmark et al., 2017), the current study examines how this PD altered teachers’ own attitudes, including their beliefs, self-efficacy and motivation for teaching. A priori, there were no expectations that other components of the affective-motivational attitudes, besides TSE, would be altered. Both qualitative and quantitative approaches were employed to analyze how science teachers in a low and middle income country (LMIC) responded to a combined neuroscience and MH PD workshop. The workshops were structured as a training-of-trainers model so that the majority of teachers would be trained by their Liberian peers, seeding local communities of practice (Westbrook et al., 2013; Kohrt et al., 2015; Chikunda, 2018). The specific research questions were:

(1) What was the fidelity of the Tier I to Tier II intervention?
(2) Did Tier I and Tier II teachers learn neuroscience sufficiently to become confident in that knowledge?
(3) How did both Tier I and Tier II trainings effect teacher affective-motivational attitudes?

## Materials and Methods

A mixed methods approach was used to assess the efficacy of a training-of-trainers PD program in neuroscience and mental health for Liberian secondary science teachers. Quantitative survey data was collected from teachers in both tiers regarding their confidence in their knowledge of neuroscience and their affective and motivational attitudes toward teaching. Qualitative data from structured interviews was collected from a subset of Tier II teachers on how they applied this knowledge in their practices.

### The Liberian Context

Liberian teachers have unmet emotional needs from traumas suffered in the civil war (1989–2003) and the Ebola epidemic (2013–15). In Liberian culture, teachers assume multiple roles beyond implementing the curriculum: acting as counselors, builders of the peace, medical personnel and psychologists (Adebayo, 2019). Administrators acknowledge these Herculean expectations but have not provided strategies or training to accomplish all these tasks (Adebayo, 2019). Education lost much of its resources, and subsequently value during the civil war. PD provides tangible, emotional support for teachers, validates their worth, and improves their skills to handle the social, emotional and intellectual needs of their students (Westbrook et al., 2013). What remains to be determined is how to deliver effective PD that combines content with training on social-emotional development and inclusive teaching practices in a low-resource environment.

Improving teacher quality through ongoing PD remains a key ingredient for addressing the educational inadequacies in Liberia (Fashina, 2017). During early post-civil war years, some teacher training programs emphasized active learning classroom strategies, but how widespread this process was or continues to be has not been documented (Barrios-Tao et al., 2017). The principle focus from international PD efforts has been on raising reading rates through elementary school teacher training (King et al., 2015; Gove et al., 2017). Examination of effective teaching practices across LMICs revealed that improving teacher communication encourages interactive pedagogies that increase student learning outcomes (Westbrook et al., 2013). Specific strategies include supporting students with feedback in a safe environment and relating content to local contexts and experiences. Group work, discussions, questioning, demonstrations, explanations, use of models and materials beyond the textbook, and use of local languages were identified as effective practices within these strategies. A key finding in this study was the need to align teacher education and PD to promote widespread use of these pedagogical practices (Westbrook et al., 2013). For secondary teachers in Liberia, the limited, available PD has been provided mostly by NGOs, with internal evaluations subject to Ministry of Education and donor requirements.

### Program Description

This pilot project was designed to train a cadre of Liberian science teachers in the neurobiology of learning and memory, emotional processing and stress, and the etiology of epilepsy and PTSD using best pedagogical practices (Darling-Hammond et al., 2017) and lessons designed for high school classrooms (Dubinsky et al., 2019). In this two tiered plan, a visiting neuroscientist delivered a 2 weeks workshop for Tier I teachers who then adopted the material for the Liberian context, delivered a series of 1 week workshops and trained additional Tier II Liberian teachers. The training-of-trainers model was selected for its ability to leverage existing local community knowledge and assets (Kutcher et al., 2016). Similar training-of-trainers models had been used in prior programs (Kohrt et al., 2018). Tier I training occurred in August 2018, and was comparable to a successful neuroscience teacher PD program in the United States (MacNabb et al., 2006b; Roehrig et al., 2012; Dubinsky et al., 2019; Schwartz et al., 2019). Lessons plans and resources used in the workshop were drawn mainly from open internet neuroscience resources (MacNabb et al., 2006a; SFN, 2019), as recommended for educational improvement in Sub-Saharan Africa (Wolfenden et al., 2012). The project represents a partnership between The Carter Center Liberia, the Ministry of Education, the Peace Corps Liberia, and local schools.

Content for both tiers included understanding the basic neuroscience of brain function and the dysfunction associated with neurological and mental disorders, modeling student-centered teaching practices including experimentation, and improving the pedagogical expertise and confidence of teachers through reflection and discussion. Tier I teachers engaged in role play and practice teaching as well. Additional content for the Tier II trainings was adopted from the Manuel of School Mental Health for Liberia (Pacione and Cooper, 2014), the Good Schools Toolkit (Devries et al., 2015) and 80 min of appropriate TedTalk and internet videos on neuroscience, recognizing and overcoming MH issues and addiction, and building relationships with students. The curricula addressed three of the five domains established for teacher PD recommended in LMICs; namely, content knowledge (neuroscience), teaching skills (modeled student-centered pedagogies) and classroom management (modeled student engagement) (Ginsburg, 2011; Pacione and Cooper, 2014). The domains of student assessment and professional ethics were not applicable to the current project (Ginsburg, 2011). Detailed content comparisons between the two tiers are presented in Results.

Eight Tier I teachers (chosen based upon knowledge, experience, availability and proximity to Monrovia) formed the Leadership Team to plan and deliver the Tier II workshops. A staff member, an Assistant Minister of Education and a Peace Corps Volunteer with 2 years teaching experience in Liberia served as co-coordinators and members of the Leadership Team. The co-coordinators coached and acted as mentors for the rest of the Leadership Team, wrote a knowledge test, and collected data. The Leadership Team delineated the content in the 5 days intensive Tier II workshops. Three Tier II workshops occurred in Monrovia and one in Kakata during fall of 2018. Based upon teacher feedback, the Leadership Team organized and ran four additional two-day follow-up Refresher sessions at local schools 3–5 months later. Refreshers included a day of practicum teaching plus a day of reflection, discussion of successes and challenges, a virtual question and answer session with the visiting neuroscientist, and collection of further data. Throughout this process, the Leadership Team discussed their own vision for the program and regularly met with representatives of the Ministry of Education for guidance.

### Participants

The Tier I workshops were attended by seventeen practicing secondary science teachers and seven master teachers working for the Ministry of Education who focused on science initiatives and training (Table 1). Ninety-two secondary science teachers attended the Tier II workshops. In keeping with Liberian traditions and as a gesture of respect and partnership, the Ministry of Education Office of Science Education selected Principals of secondary schools within the Monrovia Consolidated School System and Kakata Government Schools and requested one or two teachers be sent for training. Teachers represented a range of communities, educational backgrounds and experience (Table 1). Teacher training institutes were closed or defunct during Liberia’s civil wars, with the major teacher training institutes not graduating teachers between 1979 and 2009 (Williams, 2011). Many teachers left Liberia during the war or died. Workshop participants were representative of the current population of secondary school science teachers.

**TABLE 1:** Participant characteristics.

		Tier I	Tier II
Participants	Total	24	92
	Responded to surveys	24	61
	Male	15	63
	Female	9	29
Age	Mean (SD) range	39.5 (10.3) 23–56	35.6 (9.1) 23–62
County where live	Montserrado*	15	32
	Other	4	14
	Unknown	5	15
Education	Masters	4	1
	Bachelors	15	38
	Rural teacher training institute**	3	13
	High school	2	8
Years teaching	Mean (SD) range	9.2 (4.2) 3–20	8.5 (6.2) 2–32
Other Educational work	Yes	19	45
	Number of years	6.5 (4.4) 2–20	4.7 (3.5) 1–15
	No	5	14
Subjects taught (more than one possible)	Science	19	49
	Math	3	11
	Chemistry	1	1
	Biology	1	3
	Health	1	0
	Other	7	25
Teach in a community in a	Large city > 1,000,000	11	14
	City 100,000—1,000,000	8	28
	Town 15,000–100,000	2	15
	Village or rural area	2	2
	Unknown	1	2
Attended training on Manual of School MH***		1	1

*The capital city of Monrovia, home to one third of the country’s population, is in Montserrado County.

**Rural Teacher Training Institutes provide one-year of training for high school graduates who then proceed to teach (Ginsburg and Arrington, 2015).

***This is a separate training program on the Liberian adaptation of the WHO Manual of School Mental Health (Pacione and Cooper, 2014; Weist et al., 2017).

All participants voluntarily and formally consented to be a part of the workshop outcome study, conducted according to IRB protocols approved both by the University of Liberia and Emory University. Teachers were assigned numerical identifiers to use instead of their names on all surveys and assessments.

### Survey Instruments

Quantitative data comprised surveys teachers completed to examine changes in their knowledge and attitudes. A pre-post-delayed post design was employed. Qualitative interviews were conducted with a subset of Tier II teachers at one Refresher to further examine how teachers felt about the program and what may have changed in their teaching as a consequence. Survey scales were chosen to examine participants’ neuroscience knowledge and their confidence in teaching neuroscience content (MacNabb et al., 2006b). The affective-motivational scales of an internationally vetted instrument, the ITEL-TKS, were chosen to capture TSE and other attitudes and teacher beliefs (Sonmark et al., 2017). A few additional scales were selected from the TALIS project (OECD, 2008). Data collection was incomplete for the following scales which were not reported: TM_ESL Teacher Self Efficacy for Student Learning, TM_PD Professional Development, and TALIS 42 Classroom practices. Tier II teachers completed the surveys at the beginning and end of their workshops and on day two of the Refreshers. Tier I teachers completed the pre assessment at the end of the workshop after they had taken the post-assessment. This constituted a retrospective pre-assessment in which initial attitudes were judged relative to the final attitudes, avoiding initial overconfident self-assessment (Levinson et al., 1990; Bhanji et al., 2012).

### Quantitative Analysis

After reverse coding appropriate items, Likert scale survey responses from each of the 12 scales and their associated subscales were calculated according to the following equation: X*_*k*_* = (1/N*_*i*_*) $\sum_{i}^{N\,i}\sum_{j}^{N\,j}$*x*_*ij*_, where X*_*k*_* represents the mean score on scale *k, x_*ij*_* represents the Likert response of teacher *i* on item *j*, N*_*i*_* represents the number of teachers, and N*_*j*_* represents the number of items in scale *k*. Data from all Tier II workshops were aggregated. For all rating scales, the valence has been adjusted so that larger values represent more agreement or greater ability. Comparisons between post and retrospective pre time points for Tier I were made using two-tailed *t* tests (Graphpad Prism, version 6.1). Comparisons among pre, post and Refresher time points for Tier II data were analyzed using one-way ANOVAs followed by Tukey’s multiple comparison post-tests (Graphpad Prism, version 6.1).

### Qualitative Analysis

On the second day of one of the Refresher sessions, two Leadership Team members interviewed 10 Tier II teachers. The interviews were conducted in Liberian English using a structured interview protocol. Interview questions explored how teachers viewed their roles, student behavior and MH issues, changes, successes and challenges of their teaching and feedback on the trainings. Conversations were digitally recorded, transcribed and annotated prior to coding. Interviewers kept written field notes.

Interviews were initially coded using NVivo 12. A codebook was developed using a framework analysis approach, including an iterative reading of the interviews to generate codes (Saldana, 2016). Additional codes based on the study objectives were subsequently added. Two authors separately coded all interviews, using the field notes for context. Three authors then iteratively discussed and recoded the data until consensus was reached. Independently, two authors summarized the coded data in written form. These summaries were further discussed, edited and combined until themes emerged and consensus was reached.

## Results

### Fidelity of the Training

To assess the fidelity of the messages delivered during the Tier II workshop (research question 1), the schedules for the Tier I and Tier II workshops were compared. Considering the different activities, lectures, discussions, experiments, etc., for each, the Tier II workshops covered approximately 50% of the same material as the Tier I workshop. This includes the majority of neuroscience content, seven active learning exercises, daily reflections on the workshop and discussions of how to apply neuroscience to classroom teaching. For the neuroscience content, the Leadership Team slide presentations were edited by the Tier I instructor who answered questions and coached the presenters by email. Critical content on synaptic function and plasticity, learning and memory, neuronal circuits, cognitive function, emotional processing and decision making were similar. Lectures on brain injury and illness and epigenetics contained less mechanistic detail. Autonomic nervous system and adolescent brain development were covered in internet videos. Two Tier I content lectures had a different focus in Tier II: the introductory lecture shifted from a molecular focus to a review of neuroanatomy and neuronal structure and function. The detailed physiology of the stress response became more of an overview with practical applications regarding class disciplinary activities. The Tier II training added presentations on drugs of abuse, how to recognize behavioral and MH issues in a classroom and how to refer students to the MH clinicians (Pacione and Cooper, 2014; Devries et al., 2015). Additional internet videos illustrated recognizing and dealing with mental health needs for oneself and students through personal stories of resiliency and recovery. Two hands-on activities included in the first two Tier II workshop were subsequently dropped in the remaining Tier II workshops to provide more time for discussions. As a longer training, the Tier I workshop included 3 days of practicum teaching, three demonstration experiments, two more discussions of how to apply neuroscience to education, three additional daily reflections on workshop content, a gallery walk, a concept mapping exercise, and three review games that were not part of Tier II.

The Tier I and II workshops briefly discussed the distinctions between direct and constructivist instructional practices. Student-centered, active pedagogies were modeled in both. Teachers were asked to reflect upon the differences between what they experienced in the PD and how they themselves taught. In the practicums, teachers were given feedback on how they delivered a lesson of their choice. During the Tier II workshops and Refresher sessions, teachers openly discussed pedagogical practices.

### Confidence in Neuroscience Knowledge

**FIGURE 1:**
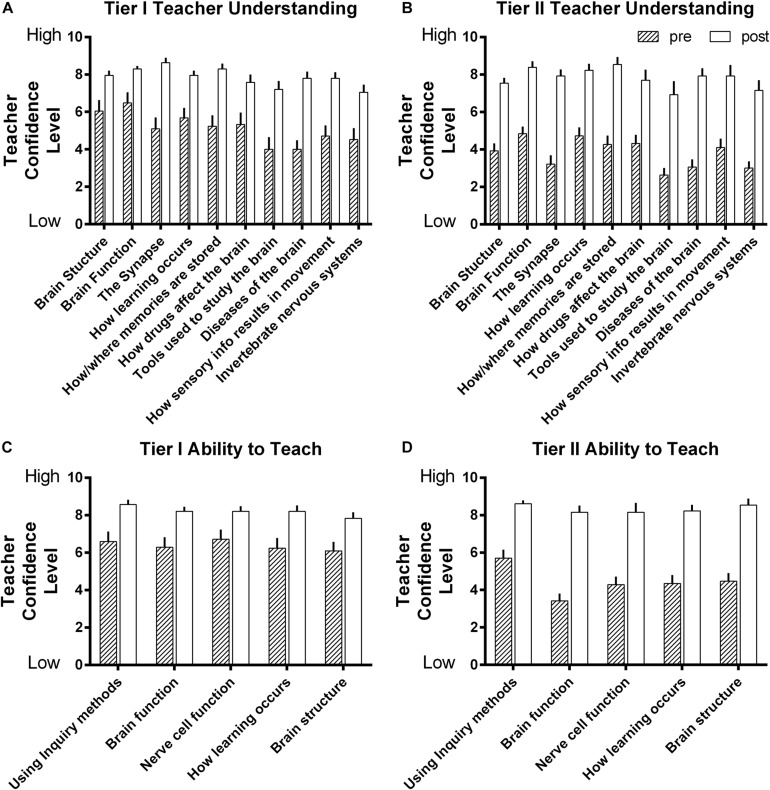
Teachers’ self-confidence ratings on their knowledge of neuroscience **(A,B)** and their ability to teach neuroscience **(C,D)**. Both Tier I **(A,C)** and Tier II **(B,D)** teachers significantly increased their self-confidence on all content items (Bonferonni post-tests all *p* < 0.05 or better after two way ANOVAs: **(A)** *p* < 0.0001 pre to post, *p* < 0.001 for item, ns interaction. **(B)** *p* < 0.0001 pre to post, *p* < 0.05 for item, ns interaction. **(C)** *p* < 0.0001 pre to post, ns for item, ns interaction. **(D)** *p* < 0.0001 pre to post, ns for item, ns interaction). Bars are mean + sem; Tier I *N* = 21 pre, 24 post, Tier II *N* = 37 pre, 13 post.

To be able to teach or apply ideas from neuroscience effectively, teachers need to be confident in that knowledge. This was achieved for both Tier I and II participants. Both Tier I and Tier II teachers demonstrated gains in neuroscience knowledge (Brick et al., 2021). Beyond this knowledge gain, teachers in both cohorts expressed an increased confidence in understanding different neuroscience concepts and their ability to teach neuroscience to others (Figure 1). Initial analysis of teacher’s daily reflections indicated that they comprehended the neuroscience content covered each day, confirming a basis for their confidence gain.

### Measures of Teachers’ Affective and Motivational Competency

Teachers in both tiers responded to an extensive survey on various aspects of affective-motivational competencies (Sonmark et al., 2017). Survey scales probed their self-efficacy, motivations for teaching, sense of responsibility and self-assessed instructional quality (Tables 2, 3).

**TABLE 2:** OECD Scales included in the Tier I teacher survey.

		Retrospective Pre-training			Post-training				
Designator	Scale and sub-scale names	Mean	SD	N	Mean	SD	N	*p*	*d*
TM_TSE	Teacher self-efficacy	59.8	19.0	23	78.6	8.6	24	**<0.0001**	1.28
TM_TSE	Student engagement	18.4	6.2	23	23.4	3.0	24	**0.0008**	1.03
TM_TSE	Instructional strategies	17.0	6.3	23	24.3	2.9	24	**<0.0001**	1.50
TM_TSE	Classroom management	19.5	6.4	23	24.7	3.1	24	**0.0010**	1.04
TM_M	Motivation to teach	59.6	18.8	23	68.5	7.8	24	**0.0382**	0.62
TM_M	Ability	15.6	5.2	23	18.1	2.7	24	**0.0452**	0.60
TM_M	Intrinsic career value	10.7	3.4	23	12.3	2.2	24	0.0544	0.58
TM_M	Extrinsic career value	12.5	5.2	23	12.1	4.7	24	0.8077	–0.07
TM_M	Social career value	20.8	7.2	23	26.0	2.8	24	**0.0054**	0.94
TM_SR	Teacher self-responsibility	52.7	16.4	23	60.0	15.3	24	0.1170	0.47
TM_SR	For student motivation	13.0	4.3	23	13.6	5.4	24	0.6854	0.12
TM_SR	For student achievement	12.4	4.3	23	13.8	4.8	24	0.3112	0.30
TM_SR	For relationships with students	13.0	5.1	23	15.7	4.2	24	**0.0490**	0.59
TM_SR	For quality of teaching	14.3	5.5	23	17.0	4.7	24	0.0755	0.53
TM_SG	Student goals (goals for teacher-student relationships)	18.3	7.4	22	21.8	5.4	24	0.0708	0.55
TM_E	Enthusiasm	10.9	3.6	22	12.8	2.3	24	**0.0359**	0.64
TM_PP	Planned persistence for teaching	10.0	3.6	22	11.7	2.3	23	0.0621	0.57
TM_WT	Willingness to invest personal time	26.1	7.8	22	29.9	5.8	24	0.0641	0.56
IQ_IQ	Instructional quality	31.2	4.2	23	32.7	4.7	24	0.2485	0.34
IQ_IQ	Monitoring	6.4	1.6	23	6.6	1.2	24	0.6427	0.14
IQ_IQ	Cognitive autonomy support for students	12.8	1.9	23	12.7	2.6	24	0.8128	–0.07
IQ_IQ	Social support for students	12.0	3.4	23	13.5	2.6	24	0.0930	0.50
IQ_CM	Classroom management	29.8	5.2	22	32.8	4.2	23	**0.0356**	0.65
IQ_UA	Understanding assessment	16.6	3.0	22	17.6	3.8	23	0.3077	0.31
TALIS 43	Dealing with disruption	12.5	2.6	19	11.4	3.3	14	0.3173	0.36
TALIS 29	Beliefs	34.7	5.7	21	36.7	5.7	24	0.2574	0.34
TALIS 29	Direct transmission beliefs about instruction	12.3	2.4	21	14.1	1.9	24	**0.0065**	0.86
TALIS 29	Constructivist beliefs about instruction	11.2	3.0	21	11.6	2.8	24	0.6923	0.12

*Subscales are indented. p-values determined by two tailed t-test comparing pre to post d, Cohen’s d, effect size.* Statistically significant p values are bolded. *Scales were developed for the ITEL-TKS or TALIS programs (Sonmark et al., 2017; OECD 2008). Higher values indicated more agreement or better abilities. The valence of the TALIS 43 scale has been reversed so that larger numbers represent improvement in handling classroom disruptions.*

**TABLE 3:** OECD Scales included in the Tier II teacher survey.

		(a) Pre-training				(b) Post-training				(c) Refresher					
Designator	Scale and sub-scale names	Mean	SD	N	a–b	Mean	SD	N	b–c	Mean	SD	N	a–c	*p*	η *2*
TM_TSE	Teacher self-efficacy	70.4	10.4	45	*	76.8	13.0	38		73.3	9.4	56		**0.0296**	0.05
TM_TSE	Student engagement	22.0	3.5	45	**	24.3	4.0	38	**	21.6	5.3	56		**0.0121**	0.06
TM_TSE	Instructional strategies	21.1	4.5	45		23.6	4.4	38		21.3	5.8	56		0.0504	0.04
TM_TSE	Classroom management	22.2	4.2	45		23.1	5.3	38		21.3	6.1	56		0.2776	0.02
TM_M	Motivation to teach	72.0	13.5	44		73.6	8.2	38	**	80.0	3.1	56	****	**<0.0001**	0.14
TM_M	Ability	17.8	3.8	44		18.7	3.2	38		20.1	1.0	56	***	**0.0004**	0.11
TM_M	Intrinsic career value	12.3	2.5	44		13.0	1.9	38		13.6	0.8	56	**	**0.0035**	0.08
TM_M	Extrinsic career value	16.0	4.9	44		15.8	4.0	38	****	19.4	1.7	56	****	**<0.0001**	0.18
TM_M	Social career value	25.5	4.4	44		26.1	3.1	38		26.9	1.5	56		0.0788	0.04
TM_SR	Teacher self-responsibility	59.5	15.1	45		65.4	13.1	37		70.7	9.4	56	****	**<0.0001**	0.13
TM_SR	For student motivation	14.1	5.6	45		15.5	4.7	37		17.3	3.2	56	**	**0.0030**	0.08
TM_SR	For student achievement	13.4	5.3	45		15.3	4.4	37		16.8	3.4	56	***	**0.0008**	0.10
TM_SR	For relationships with students	14.2	5.1	45		15.8	5.1	37		17.4	3.2	56	**	**0.0021**	0.09
TM_SR	For quality of teaching	17.7	4.0	45		18.8	3.0	37		19.3	2.3	56	*	**0.0455**	0.04
TM_SG	Student goals (goals for teacher-student relationships)	23.6	6.3	44		25.6	3.8	37		26.0	2.7	56	*	**0.0237**	0.05
TM_E	Enthusiasm	12.4	2.7	43		12.5	3.3	40		13.5	0.9	56		**0.0393**	0.05
TM_PP	Planned persistence for teaching	12.0	2.8	44		12.5	1.7	38		12.9	1.1	56		0.0516	0.04
TM_WT	Willingness to invest personal time	31.0	5.6	46		31.3	6.4	39		32.3	3.0	56		0.3763	0.01
IQ_IQ	Instructional quality	32.1	5.4	41		32.8	3.3	39		34.4	2.8	25		0.0796	0.05
IQ_IQ	Monitoring	6.1	1.5	41		5.8	1.4	39		6.1	1.1	25		0.4555	0.02
IQ_IQ	Cognitive autonomy support for students	12.9	2.9	41		12.5	2.7	39		13.9	1.7	25		0.1192	0.04
IQ_IQ	Social support for students	13.1	3.6	41	*	14.5	1.9	39		14.5	1.7	25		**0.0271**	0.07
IQ_CM	Classroom management	32.8	7.9	45		32.8	7.2	38		35.0	3.9	25		0.3797	0.02
IQ_UA	Understanding assessment	16.7	3.1	44		17.7	3.9	39		18.2	1.9	25		0.1348	0.04
TALIS 43	Dealing with disruption	11.3	2.1	44	**	12.8	2.2	38	****	9.7	1.7	56	***	**<0.0001**	0.30
TALIS 29	Beliefs	34.5	9.0	44		36.6	6.7	38		39.2	4.9	56	**	**0.0041**	0.08
TALIS 29	Direct transmission beliefs about instruction	13.0	3.5	42		12.8	3.0	38		13.4	2.2	57		0.5272	0.01
TALIS 29	Constructivist beliefs about instruction	11.6	2.5	40		11.7	2.4	38		12.6	1.7	57		0.0651	0.04

p-values determined by one-way ANOVA followed by Tukey’s post-hoc comparisons between the designated time points.

*, **, ***, **** represent p < 0.05, 0.01, 0.001, 0.0001, respectively. Statistically significant p values are bolded. *Scales were developed for the ITEL-TKS or TALIS programs (OECD, 2008; Sonmark et al., 2017). Higher values indicated more agreement or better abilities. The valence of the TALIS 43 scale has been reversed so that larger numbers represent improvement in handling classroom disruptions.*

**FIGURE 2:**
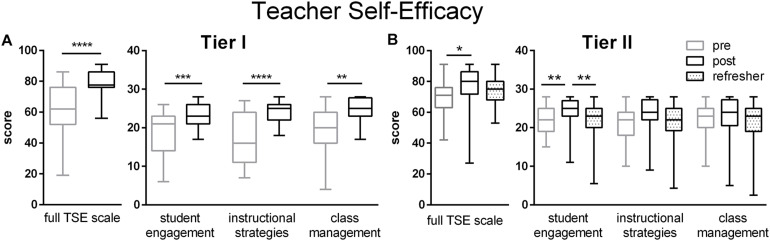
Teacher Self-Efficacy ratings for Tier I **(A)** and Tier II **(B)** teachers on both full TSE scales and subscales for Student Engagement, Instructional Strategies, and Class Management. Gains in TSE were made during the workshop for both Tier I and Tier II teachers (Tables 2, 3). Pre (gray) represent retrospective pre ratings for the Tier I teachers and pre-workshop ratings for the Tier II teachers. Boxes represent 25th to 75th percentiles with an internal bar at the median. Whiskers delineate maximum and minimum data points. Tier I *N* = 23 pre, 24 post; Tier II *N* = 45 pre, 38 post, 56 refresher.*, **, ***, and **** represent *p* < 0.05, 0.01, 0.0001, and 0.00001, respectively.

Both Tier I and Tier II teachers significantly increased their self-efficacy ratings as a result of the training (Figure 2). For Tier I teachers, this was true of all subscales (Figure 2A). For Tier II teachers, significant increases appeared for the student engagement subscale, but not the subscales for instructional strategies and classroom management (Figure 2B). In addition, at the Refresher time point for Tier II, the increased self-efficacy ratings were not sustained.

**FIGURE 3:**
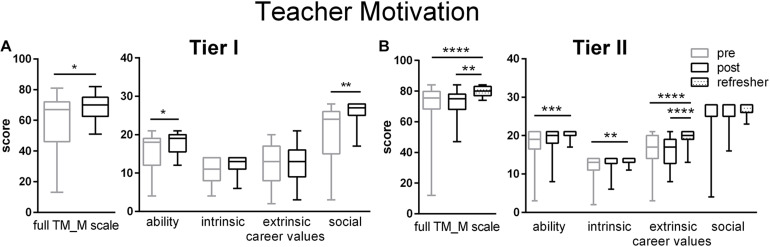
Teachers Motivation to Teach ratings for Tier I **(A)** and Tier II **(B)** teachers on both full TM_M scales and subscales for Teaching Ability, Intrinsic Career Values, Extrinsic Career Values and Social Career Values. Gains in Teacher Motivation were made more for Tier II than Tier I teachers (Tables 2, 3). Pre (gray) represent retrospective pre ratings for the Tier I teachers and pre-workshop ratings for the Tier II teachers. Boxes represent 25th to 75th percentiles with an internal bar at the median. Whiskers delineate maximum and minimum data points. Tier I *N* = 23 pre, 23 post; Tier II *N* = 44 pre, 38 post, 56 refresher.*, **, ***, and **** represent *p* < 0.05, 0.01, 0.0001, and 0.00001, respectively.

On teachers’ personal motivations to teach (TM_M scale), Tier I teachers’ ratings significantly increased at the end of the workshop but Tier II teachers’ ratings did not increase until the Refresher time point (Figure 3). Both tiers of teachers increased their self-assessment that they have the abilities and qualities of a good teacher (Ability Subscale). Changes on the personal motivation scale and subscales largely reflected a decreased variability as the ratings clustered more tightly at the top of the scales with time. This is most clearly demonstrated by Tier II teachers’ responses regarding their interest in and liking of a teaching career (Intrinsic Career Values). At the Refresher time point, Tier II teachers also felt that teaching provided a secure, stable career path (Extrinsic Career Values). After the workshop, Tier I teachers agreed more with statements regarding the ability of teachers to contribute to society by positively impacting the next generation (Social Career Values).

**FIGURE 4:**
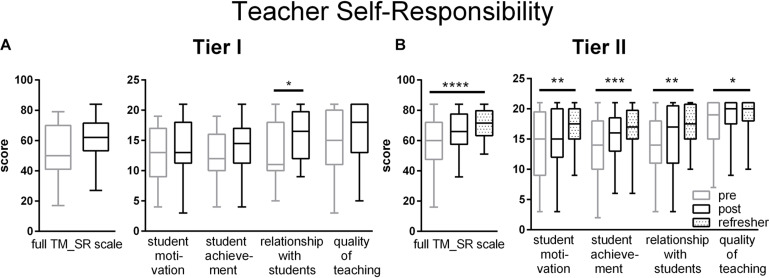
Teachers Self-Responsibility for Teaching ratings for Tier I **(A)** and Tier II **(B)** teachers on both full TM_SR scales and subscales for student motivation, student achievement, relationships with students and quality of teaching. Gains in Teacher Self-Responsibility were principally made for Tier II teachers at the Refresher time point (Tables 2, 3). Pre (gray) represent retrospective pre ratings for the Tier I teachers and pre-workshop ratings for the Tier II teachers. Boxes represent 25th to 75th percentiles with an internal bar at the median. Whiskers delineate maximum and minimum data points. Tier I *N* = 22 pre, 24 post, Tier II *N* = 41–44 pre, 38 post, 56 refresher. For the post tests, *, **, ***, and **** represent *p* < 0.05, 0.01, 0.001, and 0.0001, respectively.

One component of motivation is how teachers view their professional responsibilities regarding different aspects of their teaching, ranging from how well they motivate and interact with their students to promoting student performance through quality teaching. Changes on the scale of Teacher Self-Responsibility (TM_SR) were slow to occur. Among Tier I teachers, improvement was registered on the subscales of Relationships with Students at the end of the workshop (Figure 4A). For Tier II teachers, improvements on the full scale and all subscales occurred at the Refresher time point (Figure 4B).

Consistent with these changes in Self-Responsibility, at the Refresher time point, Tier II teachers significantly increased their ratings on their Goals for Relationships with Students (TM_SG, Table 3). After the workshop, Tier II teachers increased their ratings on their Social Support for Students (IQ_IQ, Table 3).

To address teachers’ ability to provide social and emotional support for students, the following group of scales and subscales contained questions pertinent to some aspect of student support: Self-efficacy and all 3 subscales (TM_TSE), Goals for teacher-student relationships (TM_SG), Self-responsibility for relationships with students (TM_SR subscale) and Social support for students (IQ_IQ subscale). Of these seven measures, as a group, Tier I and II teachers each changed significantly on five of them (Tables 2, 3).

At the Refresher time point, Tier II teachers registered significant increases regarding their self-responsibility for student motivation and achievement and for the quality of their teaching (Table 3). Tier I, but not Tier II, teachers significantly increased their overall enthusiasm for teaching (Tables 2, 3). Significant increases were registered on the classroom management scale by Tier I teachers and on the Dealing with Disruption scale by Tier II teachers. Tier II teachers significantly improved their overall ratings on the Beliefs scale whereas Tier I teachers’ beliefs changed only on the Direct Transmission Beliefs subscale. Tier II, but not Tier I, teachers registered a small gain in their Goals for teacher-student relationships. No changes occurred in either tier on the scales measuring their planned persistence to continue teaching, understanding of assessments, willingness to invest personal time, and the full instructional quality scales (Tables 2, 3).

### Teacher Interviews

Qualitative analysis of the Tier II teacher interviews uncovered three major themes, variously related to the affective-motivational scales. First, teachers demonstrated more confidence in their ability to reach all students and motivate them to learn, a theme that aligns with teacher self-efficacy and self-responsibility for student engagement, consistent with their ratings on the TSE and Self-responsibility scales. Second, teachers employed more student-centered pedagogical practices, an instructional strategy. Lastly, teachers increased their own emotional regulation and adapted their behaviors to prevent classroom disruption through engaging all learners, a theme aligned with TSE in the realm of classroom management.

#### Teacher Self-Efficacy and Self-Responsibility

TSE encompasses teachers’ beliefs that they can effectively engage students to learn using appropriate instructional strategies and classroom management (Tschannen-Moran and Hoy, 2001). All teachers commented upon their broadened pedagogical skills, indicating more competence and greater self-efficacy beliefs. This change in TSE was evident from their embrace of more participatory pedagogy and their decreased use of punitive discipline, as described below. Teachers felt they now had the skills to address the needs of all students, through allowing students to ask questions, discuss, and interact and through giving responsibility for learning to the student (Table 4). The definitiveness of these statements attests to the way they have internalized the instructional strategies modeled during the workshops and have incorporated them into their own practices. Teachers’ statements revealed they were able to predict student reactions when they employed these new techniques. Beyond recounting their newly adopted instructional strategies, they also commented upon their successes in student engagement and classroom management. Teachers described engaging students in the lesson through including their voice and participation in the topic to be taught; “Because by breaking them into the class discussion, you will feel a part and everybody got their own strength.”

**TABLE 4:** Pedagogical skills acquired during the trainings.

# of comments	Pedagogical skill mentioned	Example quote
19	Differentiating lessons for different level learners	“… for the fast learner, after presenting … I have to give extra curriculum. … for the normal learner …, I help them because they are already in reach, … for the slow learner, what I do, I spend much time with them, … I create extra time to go over the lesson.”
15	Putting students in groups, promoting peer learning	“I put them in group, like in one group you’ll find the fast, the normal, and the slow [learners]. In time, … every one of them starts working together.” “Maybe they will better get what they never get from me, they will be able to learn it from their colleague. So, I tell the fast learners to help the slow learners in their lesson.”
12	Class interactions and discussions	“… now my class, like … you saw this morning, is participatory” “I gave student the opportunity for them to give their view and add theirs relating to a particular topic. That makes the class very interactive.”
7	Using hands-on activities	“I try to create activities that will engage each and every student. For example, instead of just going on a board or teach, I learn that I should give hands-on activity.”
7	Assessing formatively from student explanations	“So I create challenging activity … that will require evaluation in sentences and all of them will be in a path [to learning]. At the end of the day, the objective will be met.”
5	Engaging openers	“I have little drama before we went through our topic and (with) that I can get everybody attention in the class.”
4	Encouraging student input	“… each person would have the pen, writing something, contributing toward the question. And then after everyone will come together and give their contributions.”
4	Questioning and risk taking	“I give students the opportunity to ask questions or I ask them question as to what have they learned, what do they want to learn, and what do they know as well?” “you have to encourage students to ask questions and when they would ask those questions you would be able to explain to them better.”
3	Expanding wait time	“… whenever we ask a quiz question, we should give students a breathing space for them to reflect or think about the answer. Before then, I never used to do that. I just posed my question and… I want answer right away.”
3	Gently correcting but not punishing after mistakes	“Even if he or she says the wrong thing, I will also encourage them because they have made some effort.”
3	Explaining content multiple ways	“… some of them learn by touching, by seeing, by their actions.”
1	Reflection	“I also learn to give student the opportunity to reflect their mind on past event or past topic.”
1	Asking open-ended rather than yes/no questions	“… you should ask the questions that require explanation”
1	Using analogies and making content relevant to students’ lives	“I try to take my classroom discussion to our everyday real life stuff.”
1	Giving students breaks	“I never used to allow my students to just walk out of the class [to go to the bathroom], but now I give students the chance to walk out, come back in.”

The training led to teachers taking time to analyze, reflect upon, and articulate their own roles in the classroom. The change in roles was clearly described by one teacher: “now, I feel that I have a responsibility to identify each and every one of my student[s’] needs and help them to meet the needs, so the student [is] benefiting. In the classroom, I’m not teaching student[s] to be afraid of me any longer, I’m helping them to learn. I’m facilitating their learning process, so instead of writing plenty on the board, I give activities.” In addition, teachers talked about encouraging students and building up the student’s own relationship to education and learning. “I always tell them that we the teachers, we are like people who are just there to show you the way. We are there to show you where you should go. Because the time allotted to us is not enough in class to make you understand everything. So we show you the way and I encourage my students to do further research, to go beyond what I’m giving them. So that they will better understand the subject matter.”

Teachers felt they now understood the learning process and took responsibility for the engagement and tenor of the class. Teachers attested that the new teaching skills they acquired (Table 4) engaged and motivated students to attend to class material; “we should make a student to grow up with the lesson, to invite them … to be connected with the lesson.” Teaching became an interactive process involving both students and teachers: “Instead of talking lots in class… I’m using [an] interactive method, like both students and teacher, both of us will do work together.” Capturing students’ attention and keeping them engaged avoided leaving space for annoying or disruptive behaviors to take hold.

Teachers moved from ignoring their relationships with students to focusing upon building those relationships as a means to promote learning and decrease behavioral issues in the class. An important part of building those relationships was crediting students for their contributions and participation: “I have also learned to give student credit as to what can be their input.” Teachers identified students with comprehension problems to further help those students to acquire the knowledge: “I had a student who was very difficult to talk to. … He never used to be so active. When I started showing that relationship … to him, he started pulling himself [up in class].” Teachers reached out to students to address problems while balancing the issues of equity, motivation, achievement and inclusiveness in the classroom. Beyond the classroom, teachers also strove to build relationships to help students solve personal problems that hindered student learning: “And hook yourself to the student and draw them to you by … interaction with them, …Then they too, their mind will be relaxed on your lesson or be able to grab what they will need, for your lesson.” Teachers also shared relationship-building experiences with the whole class like eating together, sharing fun, and using engaging openers. Central to the idea of many of these comments is that teacher behavior toward students can either benefit the learning or detract from it. As one teacher expressed, “For when I left [the workshop] and I went back, I had a real mindset; helping me to reach out to relate to them, when to keep quiet. How to, you know, raise my voice and even how to even … be patient with them.”

Teachers saw that the adolescent and child brain are different than adult brains, and that development of students’ brains could be influenced by teachers and education. They learned to recognize students with social, emotional, behavioral and MH problems and to engage students in addressing these problems. As one teacher recounted, “through this training…, I have noticed that as a teacher you have to observe the class and know that there are students who have mental health problems.” These educators learned to manage student misbehavior as a part of either the students’ development or their social lives outside of class. In short, teachers saw themselves as more resilient and saw students as well-rounded people with their own lives. Overall, teachers attributed their changes to understanding how the brain works: “But I think the issue of this training has brought to a fairer concept what are some of the things that we need to do in order to build a brain up and what are some of the things that are prohibited not to do with the brain.”

#### Instructional Strategies

The interviewed teachers had shifted their approach from a teacher-centered lecture or presentation to a student-centered active learning focus (Table 4). Teachers engaged students across the class intellectual and social hierarchy though use of group work, pitching the content to a level where everyone could understand, and providing additional content as needed. They included students with social, emotional, behavioral or MH problems, who previously might have been ignored or punished. Teachers paced their teaching to include everyone, not just the top students. “Mainly, my focus is to always to get at those that are not really active. Because by bringing them into the class discussion, you will feel a part and everybody got their own strength(s). Once you focusing on… the person who is always answering, you are not … doing justice to the other people.” Teachers valued contributions from the whole class, rather than seeing inactive and underachieving students as nuisances; “You make sure in the class that the slow learner will also understand the topic for that particular day. … you just stick with it, make sure that everybody in the class understands it before you move on…” Teachers seem to recognize that bringing their presentations to the level of the “slow” learner results in more learning for the whole class.

Teachers implemented peer-to-peer learning through discussions and having students work in mixed level groups (Table 4). Teachers designed lessons to focus on big ideas, relevant to students’ lives, illustrated through hands-on activities. They mentioned using group work and how it helped students to learn from each other in a new way. Skills required for group work and discussions needed to be learned and teachers seemed to appreciate this. According to one teacher, “In time, … every one of them starts working together.” Implementing group work made the lessons interactive and participatory. In the words of one teacher, “It’s good to put them in groups and get some diverse views. Then students learn from students.” The constant reference to “mixing slow and faster learners” as a post-training practice may indicate that teaching to the top students and leaving some of the class behind is a common practice. These teachers no longer considered themselves the only source of knowledge in class, but saw learning as a product of all involved: “We as teachers, we are giving them [students] these groups, we ourselves, we are learning from them, too.”

Teachers recognized they had grown in their overall understanding of learning as a biological function of the brain that requires time and practice on the part of the learner, and frequently referenced time among the challenges to implementing these changes. They allowed students time to reflect before answering a question: “Like, whenever you are teaching, you are supposed to be allotting time to students when they ask their question. You give them time, let them have relax, to respond to you.” Most importantly, teachers were cognizant that students needed time to process new information, question it and share it among peers. Changing their practice took more time to adapt the lesson for different learning abilities (”slow” and “fast”). Teachers took time after class for students and put time into those relationships. Teachers reported monitoring students over time for changes in mood or behavior as indicators that they were encountering MH problems or social issues. They acknowledged that the process of change is iterative and requires time, analysis and attention: “Because in order to analyze what I have been taught to be applied in the classroom comparing it with the old one, I need to critically analyze as to what I’m supposed to do in order to get things going well. So, these are challenges I have been faced with.”

Additional challenges included getting colleagues and students accustomed to the new teaching approaches. Abandoning the way that you were taught, as a teacher, also requires a huge leap of faith. As one teacher recounted, “Another challenge could be for the student to understand the new method I’m applying in the classroom. Sometime, a few will look at me [when I] … say, “Pupils, please sit in group.” When I tell the children, they say, “[teacher] you coming put us in our group again oo.”… So for them to agree with the change, sometime, it can be challenging. But at the end of the entire exercise, they can be happy and many of the time, they can call for more and more of the activities, so I can say even though, it’s a challenge, but it’s a pleasure to do it.” Teachers expressed concern over getting colleagues to share their new views of teaching and learning; as one teacher said, “I’m struggling with it because I want to impart that knowledge that the neuroscience training facilitators has given me. I’ve struggled with it because I want to impart that knowledge onto all of us.” When discussing these challenges, teachers demonstrated a willingness to pursue growth and change. As one teacher said, “when somebody takes step forward there will be challenges, but the ability to overcome those challenges is what matters.” Despite these difficulties, teachers viewed their changes as helpful, saying, “Even though it is challenging but I’m working [on it] … the training has made me to know those positive changes as a teacher.” Teachers also expressed concern over the lack of basics such as classroom books, resources, lab materials and even back pay, issues this training could not address.

#### Teacher Self-Regulation

Teachers recognized that their own approaches to managing their classrooms had changed. Teachers mentally connected the long term consequences of emotional and physical trauma to student learning outcomes and consequently they controlled their own behaviors to decrease any negative impact they might have on students. “As an instructor in a class sometimes a student annoys you, you get angry, you call the student up what? and slap the student’s head, which is very wrong. The workshop, the training has made us to understand that such things is wrong and believe me I decided not to practice that both home and in my school that I teach.” Teachers self-reported curtailing use of harsh disciplinary practices (Brick et al., 2021).

Teachers evolved from being aloof, vexed or openly angry at aberrant student behaviors to becoming encouraging, patient and developing good working relationships. “In the workshop, …prior to that, … anything a student does in class …I want to react. … My way of punishing them when even they are in the wrong direction [misbehaving] when I’m teaching, [now] I know the way I [will] approach them. Before then, I used to be the type of teacher who, 5 min [euphemism for “had no patience for that”], even though I was not the type to beat on students.” They described learning to control reactions, either by stopping negative reactions (profanity, temper) or by engaging positively. “I was the kind of teacher…I was very much temperamental and very restrictive, frightening students. Well, since I came to this workshop, this training, my temper dropped a little bit.” Teachers acknowledged their former role in promoting a negative classroom culture and in that acceptance gained power to now manage the classroom better. “When I went for the training, I noticed that even if [the class is] disturbing, you have a method that you would do at least to quiet the class and then you go ahead with your teaching.” All teachers emphasized the need to talk directly with students with non-compliant classroom behaviors to determine the circumstances underlying these behaviors. They preferred to talk to students one-on-one or build trust through kindness or generosity. These conversations did not occur when the student was “hot” or angry. “Firstly, if the person is behaving rude, you try to cool them down. You will not do it in the class [by saying] “shut up, stop disturbing,” no. After class, you call the student by your side, or sometimes you just giving them lunch… You will be able to cool them down and you help them calm down, at least you help them solve their mental health problems.” Thus teachers employed self-restraint and strategies to better manage the class.

## Discussion

This study examined how knowledge of the neuroscience of learning, memory, stress and emotions altered Liberian secondary science teachers affective-motivational attitudes toward their practice. Several aspects of the program were novel. This represents the first implementation of such a training program in a LMIC using a tiered training-of-trainers model. In addition, this study is the first application of an internationally constructed instrument to measure the motivational aspects of teacher competence following PD (Sonmark et al., 2017). As expected, the training-of-trainers model adapted the Tier I information to local training needs while both preserving important content and pedagogical practices and adding content on local social, emotional, behavioral and MH issues. A wide variety of teacher attitudes were observed to change either immediately or with time and practice after attending the PD. As predicted, TSE improved among both tiers of participants. Attitudes were more positive about teachers’ ability to structure lessons, engage and manage students in the process of learning. Both Tier I and Tier II teachers gained confidence in their understanding of neuroscience and ability to teach it. Surprisingly, after the workshop at the Refreshers, teachers’ motivation to teach, self-responsibility and enthusiasm had increased. In interviews, Tier II teachers commented on their new ability to reach all students and motivate them to learn, to utilize more student-centered pedagogies, and to self-regulate their own emotions to promote better classroom management, themes that align with TSE. These narrative changes represent development of their professional identities as teachers, encouraging student behaviors that promote learning, such as asking and answering questions or learning from their peers, instead of encouraging behaviors that simply lead to passing.

For both tiers, the gains in teacher knowledge, confidence in that knowledge and confidence in their ability to teach neuroscience were comparable to previously reported gains from similar workshops in a high income country (see Figures 4, 5 in MacNabb et al., 2006b). In the current setting, the Tier II teachers were instructed by their Liberian peers, the Tier I Leadership Team. This demonstrates that neuroscience knowledge can be effectively transmitted in a training-of-trainers format. Neuroscience is often considered hard, producing anxiety on the part of learners (Birkett and Shelton, 2011). However, when the neuroscience content is narrowed to concepts pertinent to learning and memory and taught using lessons designed for secondary schools, it becomes accessible to all learners (Dubinsky et al., 2019). The Tier II teacher acquisition of confidence in this knowledge demonstrated that despite expected losses of some content from shortening the workshop, teachers felt they could successfully convey the neuroscience relevant to teaching to their peers. Indeed, the adaptation of workshop content to the Liberian educational context may have accounted for the Tier II, but not Tier I, improvements on survey items related to dealing with disruption, teacher self-responsibility, student achievement and student relationships.

### Teacher Self Efficacy

TSE refers to teachers’ beliefs regarding their ability to produce student learning, i.e., the personal ability to provide appropriate and meaningful instruction and the outcome ability to achieve appropriate student growth and performance (Bandura, 1997). Personal self-efficacy included teachers’ self confidence that they have mastered the subject matter sufficiently and had the confidence to teach it appropriately to student audiences (Bandura, 1997). The instrument operationalizing assessment of TSE recognizes that resources and environments constrain practices and focuses upon activities normally encountered in teachers’ work: student engagement, instructional strategies, and classroom management (Tschannen-Moran and Hoy, 2001; Sonmark et al., 2017). In a review summarizing 40 years of TSE research, TSE has been positively linked to measures of teachers own well-being, personal accomplishment, job satisfaction, and commitment (Zee and Koomen, 2016). TSE also positively influences many aspects of teachers’ practices, including employing effective and innovative learning strategies, connecting to students’ lives, providing social and emotional support for students, classroom management, differentiation, and inclusivity (Zee and Koomen, 2016). Overall, high TSE is associated separately with greater use of constructivist, student-centered instructional approaches, and better academic achievement (Zee and Koomen, 2016). Across national boundaries and collectivist vs. individualist cultures, teachers with higher self-efficacy reported more productive teaching practices and higher job satisfaction (Klassen and Chiu, 2010; Vieluf et al., 2013). PD frequently leads to increased TSE (Zee and Koomen, 2016), as also demonstrated here. Similarly, PD in inquiry science teaching involving active participation by teachers, reflection, and follow-up increased their self-efficacy for specifically teaching scientific inquiry (Lotter et al., 2018). Here, teachers’ confidence in their neuroscience knowledge reflected their “cognitive mastery” of that knowledge, a critical part of self-efficacy (Palmer, 2006). Liberian teachers’ new ability to engage students, use student-centered practices and manage their classrooms more effectively were a major departure from their previous beliefs and practices, as revealed in the surveys and interviews, and constitute improvements in their TSE.

At follow-up, the relaxation of TSE attitudes to initial levels attests to the difficulty of sustaining new beliefs and practices in the absence of adequate support. A similar reversal at follow-up of end of workshop gains in self-efficacy for inquiry teaching were reported in a pilot study of US middle school science teachers (Lotter et al., 2016). When the samples size was increased, a sustained increase in self-efficacy for inquiry teaching was observed, indicating that weak elements of a pilot intervention can be subsequently corrected (Lotter et al., 2018). Alternatively, by the time teachers had been implementing new classroom practices for several months, they may have reset their internal assessment of their own capabilities. Future studies should administer a retrospective pre-survey at the same time as the follow-up survey, so that both reflect teacher’s internal ratings on the same day. Retrospective pre-tests can be more accurate assessments of prior knowledge since one doesn’t realize the extent of initial ignorance until after learning the new material (Levinson et al., 1990; Bhanji et al., 2012). In addition, more, continuous follow-up support may be needed to solidify the initial TSE gains.

Interviewee comments on instructional practices clearly favored a more student-centered approach. Without observing participants in their classrooms, interpretations of the Beliefs scale become difficult. Tier I ratings of statements associated with direct instruction increased while Tier II ratings trended toward increasing constructivist beliefs. The individual questions on this scale that showed change were “Effective/good teachers demonstrate the correct way to solve a problem” and “Instruction should be built around problems with clear, correct answers, and around ideas that most students can grasp quickly,”(OECD, 2008). Even in a constructivist-oriented classroom, having a teacher summarize by providing a correct interpretation or solution, is an excellent practice. Similarly, initially focusing students on doable problems so that they succeed and gain confidence provides scaffolding necessary for subsequent deeper or open-ended challenges. In the absence of direct assessment of these teachers’ pedagogical practices before and after the workshop, these survey results should be interpreted with caution.

The qualitative impacts of the training on teacher’s emotional regulation and classroom management were unexpected, as these aspects of teaching had not been intentionally targeted in the trainings. Altering teachers’ views of student behavior and potential to learn may have provided them with the patience to approach behavioral problems from a more tolerant and less stressful perspective. Unexpected impacts upon classroom management have previously been reported from interventions targeting lowering teacher stress through mindfulness and social-emotional skills trainings (Jennings et al., 2017). Changing classroom management strategies reflects a major shift in participants’ thinking. Culturally, maintaining classroom discipline is highly valued by Liberians, being the second strongest reason parents cite for choosing a school, after teacher quality (Longfield and Tooley, 2017). In the interviews, teachers indicated they maintained better control of their own emotions when responding to student misbehaviors, a form of self-regulation which is also linked to professional competence (Klusmann et al., 2008; Kunter et al., 2013). Self-regulation was not directly assessed in the extensive ITEL-TKS instrument. While the interviews indicated management strategies shifted toward promoting student engagement, the survey results did not uniformly reflect such changes. The absence of change in the Tier II classroom management ratings may represent the fact that teachers who employ student-centered practices often do not have as much control over classroom behaviours (Owens and Tanner, 2017). Consistent with interviewee’s reports of allowing more discussion and group work, Tier II ratings decreased on the Dealing with Disruptions scale. Questions on this scale address noise levels, classroom interruptions and getting students to quiet down, behaviors that would be expected to increase with more student-centered practices (Owens and Tanner, 2017). The Liberian teacher testaments to using both group work and a more positive classroom climate parallel recommendations for effective pedagogy in LMIC (Westbrook et al., 2013).

Gains in the Liberian teachers’ skills for self-awareness, emotional regulation, and building teacher-student relationships parallel three of the five competencies recognized for effective social and emotional learning (Schonert-Reichl et al., 2017). For adults and children, SEL concerns the processes for developing social and emotional competencies for self-awareness, social awareness, responsible decision making, self-management and relationship management (Schonert-Reichl et al., 2017). To promote better student learning of social-emotional skills, teachers must be supported in developing their own social-emotional competencies (Jones and Kahn, 2017). Teachers with better social-emotional skills engage more with their students, build stronger positive relationships and engage in better classroom management (Jones and Kahn, 2017). Teachers in comprehensive skill building programs learned how to recognize, understand and regulate their own emotions and demonstrated more positive teacher-student interactions, responses to emotions and caring beyond the classroom (Brackett et al., 2019). Providing the neuroscientific basis for how emotions and stress influence learning and memory in conjunction with discussions of effective teaching and how to recognize student emotional issues in the Liberian program appeared to produce comparable results. While the pillars of social-emotional competency programs (Osher et al., 2016) were not specifically taught here, combining those principles with a neuroscientific foundation for learning and memory may enhance effectiveness of future programs.

### Motivations for Teaching

The improvements in motivations for teaching for both tiers of teachers was not expected. Teachers’ motivation for teaching includes their own professional goals, sense of responsibility, and enthusiasm as well as their psychological needs. Most importantly, teacher motivation is related to their pedagogical knowledge and to their decisions to choose and implement high-quality pedagogy (Konig and Rothland, 2012). Teachers’ motivation and goals predict their professional learning and subsequent practice (Thoonen et al., 2011; Nitsche et al., 2013). While teacher motivation can positively influence participation in PD (Lauermann et al., 2017) and subsequent implementation of that PD content (Gaines et al., 2019; Osman and Warner, 2020), whether PD can alter teacher motivation has not been widely addressed (Saunders, 2013). Pre-service teachers’ initial motivations for teaching are positively associated with their practices at induction (Richardson and Watt, 2014). Changes in pre-service teachers’ motivations to teach over the course of their training and induction have been documented. Improvements in teacher motivation can occur when they gain or exercise agency over some aspect of their practice or take on leadership roles (Han and Hongbiao, 2016). Frustrations associated with acquisition of new knowledge can also be demotivating (Han and Hongbiao, 2016) so predictions regarding the impact of PD on teacher motivation are hard to ascertain. The current study suggests that motivations to teach are malleable, even among seasoned teachers, when PD provided new conceptualizations of how learning occurs combined with introductions to constructivist practices.

Teachers’ personal and situationally driven motivations may vary according to the larger social or school specific contexts. In an international comparison of the development of mathematics teacher knowledge, an intrinsic interest in math increased motivation to invest time and energy and overcome difficulties, whereas an extrinsic goal to achieve job security decreased that motivation (Blomeke and Delaney, 2012). Among pre-service teachers in the US, intrinsic and social motivations mediate teacher self-responsibility, TSE, interest in PD, personal time investment, and commitment to teaching as a career (Lauermann et al., 2017). In the Liberian context, Tier II teachers registered increases in the motivational subscales of intrinsic, extrinsic and ability values. Tier I teachers registered increases in the subscales of ability and social career values. Neither tier reported changes in willingness to invest personal time. If replicable, the reasons behind these context-specific changes require further investigation.

Liberian teachers demonstrated their persistently high motivation and sense of personal responsibility to improve educational outcomes, despite the economic and structural adversities encountered in their country. Teachers’ pay was delayed or not received in the interval between the workshop and the Refresher sessions, a common occurrence and factor that works against professional commitments (Adebayo, 2019; Johnson, 2019; Romero and Sandefur, 2019), resulting in strikes and student protests (Dunbar, 2019). Despite this issue and other structural problems, teacher motivation to teach and self-responsibility for student outcomes increased between the workshop and the Refreshers. These results are in contrast to reported de-motivating outcomes for PD in Malawi where similar structural problems of low or absent pay and empty government promises undermined change (Selemani-Meke, 2013).

### Self-Responsibility

Teacher self-responsibility represents what they feel they should be doing in contrast to what they feel they can do (TSE) (Lauermann and Karabenick, 2013). While correlations exist between responsibility and efficacy for each of the subscale factors (student motivation, student achievement, relationships with student, and teaching), the self-responsibility scales capture distinct dimensions of affective-motivational attitudes (Lauermann and Karabenick, 2013). Self-responsibility predicts TSE and interest in PD (Lauermann, 2017). Self-responsibility has generally been examined among teachers at a single timepoint (Lauermann, 2017). Like motivation, how self-responsibility may change following PD has not been previously reported. The change registered in Liberian teachers’ sense of self-responsibility was toward forming more supportive relationships with learners. Tier II teachers sense of responsibility for quality of teaching, student motivation and student achievement also increased at the follow-up time point. Following the current training, participants embraced the modeled pedagogies as a means for engaging students and providing social and emotional support. Among high school teachers, embracing a growth mindset view of their students predicts teacher self-responsibility and both predict adoption of mastery practices (Matteucci et al., 2017). Student performance improves following relatively short instruction in the neuroscience of learning and memory linked to ideas promoting a growth mindset (Blackwell et al., 2007; Yeager et al., 2019). Thus, it is plausible that teachers’ views of their students’ potential also shifted following more intensive neuroscience PD. In addition to teachers’ expected roles as content expert, deliverer of quality teaching and role model, Liberian teachers view their positions as also encompassing parenting and counselling (Adebayo, 2019). The changes reported here suggest a deepened commitment of the teachers toward these altruistic goals and a better understanding of how to achieve them as a consequence of their deeper knowledge of students gained during the workshops. The conjoint positive changes in TSE and self-responsibility exemplify theoretical predictions stating that optimistic personal expectations together with opportunities for personal growth should foster more responsibility, even in the face of adverse outcomes (Lauermann and Karabenick, 2011).

### Impact of Neuroscience

Neuroscience provided two messages that the teachers embraced. Understanding synaptic plasticity provided a new view of the ability of all students to learn. This idea motivated teachers to adopt more student-centered practices despite large class sizes, limited space and little on-the-ground support. Gaining insight into normal brain growth, development, learning and the neurophysiology of social, emotional and behavioral disorders motivated teachers to change their own behaviors. Their own motivations to increase student engagement became stronger and they applied novel teaching strategies not yet widely practiced in West Africa (Westbrook et al., 2013).

Another strong realization among the interviewed teachers was that the stress felt by students on the receiving end of negative reinforcement was a neurophysiological detriment to their being able to learn. Teachers understood that the presence of stress hormones inhibited brain circuits for learning. Recognizing that producing such stress through shaming or punishment was antithetical to their goals for student learning, teachers opted to improve their self-control. This was an unexpected, but welcome outcome that programs in other countries with such problems may want to replicate (Antonowicz, 2010). Both outcomes demonstrated ways that the neuroscience content provided knowledge of students that teachers utilized in their daily interactions. Their ability to apply this knowledge was facilitated by the content added in Tier II addressing how to recognize student social, emotional and behavioral issues. Moreover, the training process, content and subsequent changes in practice improved TSE, motivation and self-responsibility for student relationships and success, all aspects of the affective-motivational dimension of teacher competence.

The Liberian neuroscience training demonstrated how understanding basic neuroscience concepts in combination with discussions of students’ social, emotional and MH needs may change teachers’ affective-motivational attitudes toward students and their practice. For pre-service teachers, the productive friction that occurs when views are challenged acts as a factor driving motivational change (Nolen et al., 2014). Understanding the neuroscience of learning, memory, emotions and stress may have produced such productive friction in the Liberian teachers. Returning to Shulman’s conception, the knowledge base for teaching is not fixed or final but should grow with insights from research (Shulman and Shulman, 2004). The development of a detailed neuroscientific understanding of the biological basis for learning and memory within the past 50 years is now ripe for inclusion into teacher training. The Science of Learning incorporates neuroscience into education, reflecting this dynamic view of teacher knowledge (Meltzoff et al., 2009; Ansari et al., 2017; Revai and Guerriero, 2017). Teachers should be able to reflect, incorporate new understandings, and learn from research as well as experience (Shulman and Shulman, 2004). That is what happened in the Liberian program. Challenging teachers to apply this new knowledge of students stimulated them to change their approach toward interacting with students, working to build relationships, motivating and engaging all learners.

### Structural Elements That Made the Program a Success

Core components that contributed to the success of the program included the local adaptation of the content, focus on current science teachers, and a Leadership Team that was tasked with executing the model. Rather than impose a top-down training, the Tier II workshops were geared to the local contexts, a practice recommended over policies derived from different contexts (Pritchett and Sandefur, 2013). The local adaptation of the original content and schedule was critical. Going through the process of identifying the important big ideas focused the Leadership Team. Staff helped the Leadership Team remain on task. Additionally, dividing up the neuroscience content among Leadership Team members also lowered the initial barrier to teach this content. Being part of a team strengthened individuals’ confidence to be able to share this knowledge with the Tier II audiences. The Leadership Team was motivated by the honor of being included and by their own certainty that the content was important to share with their colleagues.

While implementation fidelity has been an aspirational goal in scaling up programs, a balance must be achieved for accommodating adaptation to the local conditions, as described here (Perez et al., 2016). Among LMIC educational reforms, matching pedagogy to local students’ levels and needs provides a cost-effective means of improving learning outcomes, especially when programs are tailored to local conditions (Kremer et al., 2013). Recognizing the adaptive nature of the training-of-trainers process, this study focused primarily on the Tier II teacher outcomes. Tier I teacher outcomes are reported to demonstrate that those teachers who became part of the Leadership Team did indeed learn and understand the delivered Tier I content and were therefore capable of transmitting that knowledge to the Tier II teachers. One benefit of a training-of-trainers structure for teacher PD is the empowerment of Liberian teachers to propagate the change messages. Their agency built local leadership as well as modeled problem solving behaviors for other teachers. Moreover, the local control worked to diminish the perceived power of external funders over the minimal capacity of local institutions (Open Society Foundations, 2015).

### Policy Implications

A meta-analysis of the cost effectiveness of various interventions on improving education in LMIC found that providing more effective pedagogy increased test scores more than simply lowering class sizes (Kremer et al., 2013). Combining neuroscience and MH training for Liberian teachers using this training-of-trainers PD model, providing knowledge of how students learn, would therefore be an effective strategy for improving students’ educational experiences. The next step in this process would be to formalize current agreements with local teacher training institutions and the Ministry of Education to include neuroscience and social, emotional, behavioral and MH issues in their pre-service and in-service curriculum for teachers. With more universally trained teachers, the neuroscience knowledge of how learning occurs could be transferred to students, where a growth mindset might be promoted (Yeager et al., 2019). Additional benefits that might be expected (and could be studied) would be increasing student motivations to complete their schooling, performance on exit exams, and/or interest in science.

### Limitations

All of the data presented here, both surveys and interviews, are teacher self-reports. The limited resources for this pilot program did not permit active observation of classrooms. A number of scales from the ITEL-TKS and the previous TALIS program (OECD, 2008; Sonmark et al., 2017) captured instructional choices, beliefs, management and assessment methods. With the large number of statistical comparisons, two or three significant changes would be expected by chance alone. These self-reports could reflect an incremental change in practice or a report of an intention to change. Since the interviewees were all volunteers, the oral reports may have captured opinions from only the most ardent proponents of change. Future in depth studies should include classroom observations to verify and provide support for enacting changes in teacher practices.

Many of the attitude changes reported for Tier II teachers did not occur until the Refresher time point. Attitudinal changes registered at the end of a 1 or 2 weeks training would not be considered to have withstood the test of time. However, for changes to be registered at the Refresher time point, after teachers had had time to implement ideas encountered in the training, testifies to the lasting effects that can accrue from short interventions.

Since the program intertwined neuroscience and MH content with discussions and modeling of best pedagogical practices, outcomes cannot be attributed to the neuroscience alone. Indeed, PD combining content knowledge with pedagogy produces better student and teacher outcomes than PD focused on content alone (Roth et al., 2019). On an international scale, other PD programs have similarly reported pedagogical improvements, with more enjoyment of school, more acceptance of student-centered pedagogy, and more positive attitudes toward regular students but not those with perceived disabilities (Westbrook et al., 2013). Future programs should consider including an active control group receiving pedagogical training for comparison.

## Conclusion

The Liberian PD provided teachers with knowledge of the neuroscience of learning, memory, stress and emotions using student-centered pedagogy combined with training on recognizing students’ social, emotional, behavioral and MH issues. In experiencing the workshop content as students would, teachers were able to see themselves as learners, identify with students’ needs, and apply some of the social and emotional messages to their own lives as teachers. The Liberian teachers demonstrated an increased self-awareness of their emotional responses to misbehaving students. They reported controlling their emotions in those situations and making appropriate decisions regarding responses, helping to build teacher-student relationships.

The current results demonstrated that PD in neuroscience and MH has the capacity to build teacher self-efficacy, motivation, self-responsibility and other affective-motivational attitudes characteristic of competent teachers. These attitudes, measured on an internationally vetted instrument, are malleable. Consistent with the changes in attitudes, teachers self-reported an increased ability to engage and motivate learners, utilize student-centered pedagogies, and control their own emotions when managing their classes. Including neuroscience content into educator training provides teachers with necessary, foundational knowledge of students - how they learn and mature intellectually and how life experiences can support or undermine those processes.

## Data Availability Statement

The raw data supporting the conclusions of this article will be made available by the authors, without undue reservation.

## Ethics Statement

All participants voluntarily and formally consented to be a part of the workshop impact study, conducted according to IRB protocols approved separately by the University of Liberia and Emory University. The patients/participants provided their written informed consent to participate in this study.

## Author Contributions

KB managed the program, developed curriculum, delivered the Tier II trainings, curated, analyzed, interpreted data, and edited the manuscript. JLC conceived of, designed and led the program and evaluation, developed curriculum, analyzed, interpreted data, and edited the manuscript. LM managed the program, developed curriculum, and delivered the Tier II trainings. SF managed the program, developed curriculum, and delivered the Tier II trainings. JM curated and analyzed quantitative data. JMD designed the curriculum, Tier I trainings and evaluation, delivered the Tier I trainings, provided the neuroscience content, analyzed and interpreted data, wrote, and edited manuscript. All authors contributed to the article and approved the submitted version.

## Conflict of Interest

The authors declare that the research was conducted in the absence of any commercial or financial relationships that could be construed as a potential conflict of interest.

## References

[B1] Adebayo S. B. (2019). *Emerging Perspectives of Teacher Agency in a Post-Conflict setting: The case of Liberia. Teaching and Teacher Education [Online], 86.* Available online at: 10.1016/j.tate.2019.102928 (accessed Febuary 2, 2021).

[B2] African Development and Bank (2020). *African Economic Outlook 2020.* Abidjan: African Development Bank.

[B3] Ahmad S. I. Abubakar B. B. Yau S. (2018). Biology education a panacea for sustainable national development. *Front. Environ.l Microbiol.* 4:71–74. 10.11648/j.fem.20180402.14

[B4] Ansari D. Konig J. Leask M. Tokuhama-Espinosa T. (2017). “Developmental cognitive neuroscience: implications for teachers’ pedagogical knowledge,” in *Pedagogical Knowledge and the Changing Nature of the Teaching Profession*, ed. Guerriero S. (Paris: OECD Publishing), 195–222. 10.1787/9789264270695-11-en

[B5] Antonowicz L. (2010). *Too Often in Silence. A Report on School-Based Violence in West and Central Africa”. P.W.A. Save the Children.* New York, NY: UNICEF.

[B6] Bandura A. (1997). *Self-efficacy: The Exercise of Control.* New York, NY: Freeman.

[B7] Barrios-Tao H. Siciliani-Barraza J. M. Bonilla-Barrios B. (2017). Education programs in post-conflict environments: a review from liberia, sierra leone, and South Africa. *Rev Electrón Educare* 21:1. 10.15359/ree.21-1.11

[B8] Baumert J. Kunter M. (2013). “The COACTIV model of teachers’ professional competence,” in *Cognitive Activation in the Mathematics Classroom and Professional Competence of Teachers, Mathermatics Teacher Education*, eds Kunter M. Baumert W. Blum U. Klusmann S. Krauss M. N. (New York, NY: Springer Science+Business Media). 10.1007/978-1-4614-5149-5_1

[B9] Bhanji F. Gottesman R. De Grave W. Steinert Y. Winer L. R. (2012). The retrospective pre–post: a practical method to evaluate learning from an educational program. *Acad. Emerg. Med.* 19 189–194. 10.1111/j.1553-2712.2011.01270.x 22320369

[B10] Birkett M. Shelton K. (2011). Decreasing neuroscience anxiety in an introductory neuroscience course: an analysis using data from a modified science anxiety scale. *J. Undergrad. Neurosci. Educ.* 10 A37–A43. 23626491 PMC3598191

[B11] Blackwell L. S. Trzesniewski K. H. Dweck C. S. (2007). Implicit theories of intelligence predict achievement across an adolescent transition: a longitudinal study and an intervention. *Child Dev.* 78 246–263. 10.1111/j.1467-8624.2007.00995.x 17328703

[B12] Blomeke S. (2017). “Modelling teachers’ professional competence as a multi-dimensional construct,” in *Pedagogical Knowledge and the Changing Nature of the Teaching Profession*, ed. Guerriero S. (Paris: OECD Publishing), 119–172. 10.1787/9789264270695-7-en

[B13] Blomeke S. Delaney S. (2012). Assessment of teacher knowledge across countries: a review of the state of research. *ZDM Math. Educ.* 44 223–247. 10.1007/s11858-012-0429-7

[B14] Brackett M. A. Bailey C. S. Hoffmann J. D. Simmons D. N. (2019). RULER: a theory-driven, systemic approach to social, emotional, and academic learning. *Educ. Psychol.* 54 144–161. 10.1080/00461520.2019.1614447

[B15] Brick K. Cooper J. L. Mason L. Faeflen S. Monmia J. Dubinsky J. M. (2021). *Training-of-Trainers Neuroscience and Mental Health Teacher Education in Liberia.* 10.3389/fnhum.2021.653069 PMC8249721 34220469

[B16] Chikunda C. (2018). *Philosophies, Theories and Principles for ESD in Teacher Education,” in Guidebook on Education for Sustainable Development for Educators. Effective Teaching and Learning in Teacher Education Institutions in Africa 2018.* Paris: UNESCO Digital Library, 45–62.

[B17] Darling-Hammond L. Hyler M. E. Gardner M. (2017). *Effective Teacher Professional Development.* Palo Alto, CA: Learning Policy Institute.

[B18] Devries K. M. Knight L. Child J. C. Mirembe A. Nakuti J. Jones R. (2015). The good school toolkit for reducing physical violence from school staff to primary school students: a cluster-randomised controlled trial in Uganda. *Lancet Global. Health* 385 e378–e386. 10.1016/S2214-109X(15)00060-1 PMC4928210 26087985

[B19] Dommett E. J. Devonshire I. M. Plateau C. R. Westwell M. S. Greenfield S. A. (2011). From scientific theory to classroom practice. *Neuroscientist* 17 382–388. 20484219 10.1177/1073858409356111

[B20] Dubinsky J. M. Guzey S. Schwartz M. S. Roehrig G. Macnabb C. Schmied A. (2019). Contributions of neuroscience knowledge to teachers and their practice. *Neuroscientist* 25 394–407. 10.1177/1073858419835447 30895863

[B21] Dubinsky J. M. Roehrig G. H. Varma S. (2013). Infusing neuroscience into teacher professional development. *Educ. Res.* 42 317–329. 10.3102/0013189x13499403 26139861 PMC4485447

[B22] Dunbar A. (2019). *Liberia: MCSS Students Block Presidential Convoy In Massive Protest Over Teachers’ Salaries; Finance Ministry Says Pay on the Way.* Available online at: https://frontpageafricaonline.com/news/liberia-mcss-students-block-presidential-convoy-in-massive-protest-over-teachers-salaries-finance-ministry-says-pay-on-the-way/ (accessed October 16, 2019).

[B23] Education 2030 (2018). *Guidebook on Education for Sustainable Development for Educators. Effective Teaching and Learning in Teacher Education Institutions in Africa.* Paris: UNESCO.

[B24] Fashina A. A. (2017). Teacher quality and liberia’s educational system. arts and humanities. *Open Access. J.* 1 132–133. 10.15406/ahoaj.12017.15401.00022

[B25] Gaines R. E. Osman D. J. Maddocks D. L. S. Warner J. R. Freeman J. L. Schallert D. L. (2019). Teachers’ emotional experiences in professional development: where they come from and what they can mean. *Teach. Teach. Educ.* 77 53–65. 10.1016/j.tate.2018.09.008

[B26] Ginsburg M. (2011). *EQUIP2 State-of-the-Art Knowledge in Education. Teacher Professional Development. A Guide to Education Project Design Based on a Comprehensive Literature and Project Review.* Washington DC: USAID.

[B27] Ginsburg M. Arrington B. (2015). “Diverse partnerships: designing and implementing the Liberia Teacher Training Program,” in *Partnership Paradox. The Post-Conflict Reconstruction of Liberia’s Education System*, eds Talbot C. Taylor A. (New York, NY: Open Society Foundations), 165–180.

[B28] Gove A. Korda Poole M. Piper B. (2017). “Designing for scale: Reflections on rolling out reading improvement in Kenya and Liberia,” in *Progress Toward a Literate World: Early Reading Interventions in Low-Income Countries*, eds Gove A. Mora A. Mccardle P. (New York, NY: Wiley). 10.1002/cad.20195 28267285

[B29] Guerriero S. (2017a). *Pedagogical Knowledge and the Changing Nature of the Teaching Profession.* Paris: OECD Publishing.

[B30] Guerriero S. (2017b). “Teachers’ pedagogical knowledge: What it is and how it functions,” in *Pedagogical Knowledge and the Changing Nature of the Teaching Profession*, ed. Guerriero S. (Paris: OECD Publishing), 99–118. 10.1787/9789264270695-en

[B31] Guerriero S. Revai N. (2017). “Knowledge-based teaching and the evolution of a profession,” in *Pedagogical Knowledge and the Changing Nature of the Teaching Profession*, ed. Guerriero S. (Paris: OECD Publishing), 253–269.

[B32] Han J. Hongbiao Y. (2016). Teacher motivation: definition, research development and implications for teachers. *Cogent. Educ.* 3:1217819. 10.1080/2331186X.2016.1217819

[B33] Howard-Jones P. A. Jay T. Galeano L. (2020). Professional developmenton the science of learning and teachers’ performative thinking – a pilot study. *Mind, Brain Educ.* 14 267–278. 10.1111/mbe.12254

[B34] Jennings P. A. Brown J. L. Frank J. L. Doyle S. Oh Y. Davis R. (2017). Impacts of the CARE for teachers program on teachers’ social and emotional competence and classroom interactions. *J. Educ. Psychol.* 109 1010–1028. 10.1037/edu0000187

[B35] Johnson O. (2019). *Liberia Anti-Corruption Commission Comptroller Resigns, Says Working without Pay for Months Makes No Sense. Front Page Africa.* Availablre online at: https://frontpageafricaonline.com/news/liberia-anti-corruption-commission-comptroller-resigns-says-working-without-pay-for-months-makes-no-sense/ (accessed November 26, 2019).

[B36] Jones S. M. Kahn J. (2017). *The Evidence Base for How We Learn. Supporting Students’ Social, Emotional, and Academic Development.* Washington, DC: The Aspen Institute.

[B37] King S. Korda M. Nordstrum L. Edwards S. (2015). *Liberia Teacher Training Program: Endline Assessment of the Impact of Early Grade Reading and Mathematics Interventions.* Research Triangle Park, NC: USAID/RTI International.

[B38] Klassen R. M. Chiu M. M. (2010). Effects on teachers’ self-efficacy and job satisfaction: teacher gender, years of experience, and job stress. *J. Educ. Psychol.* 102 741–756. 10.1037/a0019237

[B39] Klusmann U. Kunter M. Trautwein U. Ludtke O. Baumert J. (2008). Teachers’ occupational well-being and quality of instruction: the important role of self-regulatory patterns. *J. Educ. Psychol.* 100 702–715. 10.1037/0022-0663.100.3.702

[B40] Kohrt B. A. Blasingame E. Compton M. T. Dakana S. F. Dossen B. Lang F. (2015). Adapting the Crisis Intervention Team (CIT) model of police-mental health collaboration in a low-income, post-conflict country: curriculum development in Liberia, West Africa. *Am. J. Public Health* 105 e73–e80. 25602903 10.2105/AJPH.2014.302394 PMC4330847

[B41] Kohrt B. A. Mutamba B. B. Luitel N. P. Gwaikolo W. Onyango M. P. Nakku J. (2018). How competent are non-specialists trained to integrate mental health services in primary care? Global health perspectives from Uganda, Liberia, and Nepal. *Int. Rev. Psychiatry* 30 182–198. 10.1080/09540261.2019.1566116 30810407 PMC6499679

[B42] Konig J. Rothland M. (2012). Motivations for choosing teaching as a career: effects on general pedagogical knowledge during initial teacher education. *Asia Pacif. J. Teach. Educ.* 40 289–315. 10.1080/1359866x.2012.700045

[B43] Kremer M. Brannen C. Glennerster R. (2013). The challenge of education and learning in the developing world. *Science* 340 297–300. 10.1126/science.1235350 23599477

[B44] Kunter M. Klusmann U. Baumert J. Richter D. Voss T. Hachfeld A. (2013). Professional competence of teachers: effects on instructional quality and student development. *J. Educ. Psychol.* 105 805–820. 10.1037/a0032583

[B45] Kutcher S. Wei Y. Gilberds H. Ubuguyu O. Njau T. Brown A. (2016). A school mental health literacy curriculum resource training approach: effects on Tanzanian teachers’ mental health knowledge, stigma and help seeking efficacy. *Int. J. Ment. Health Syst.* 10:50. 10.1186/s13033-13016-10082-13036 PMC4973111 27493684

[B46] Lauermann F. (2017). “Teacher motivation, responsibility, pedagogical knowledge and professionalism: a new era for research,” in *Pedagogical Knowledge and the Changing Nature of the Teaching Profession*, ed. Guerriero S. (Paris: OECD Publishing), 171–191.

[B47] Lauermann F. Karabenick S. A. (2011). Taking teacher responsibility into account(ability): explicating its multiple components and theoretical status. *Educ. Psychol.* 46 122–140. 10.1080/00461520.2011.558818

[B48] Lauermann F. Karabenick S. A. (2013). The meaning and measure of teachers’ sense of responsibility for educational outcomes. *Teach. Teach. Educ.* 30 13–26. 10.1016/j.tate.2012.10.001

[B49] Lauermann F. Karabenick S. A. Carpenter R. Kuusinen C. (2017). “Teacher motivation and professional commitment in the united states the role of motivations for teaching, teacher self-efficacy and sense of professional responsibility,” in *Current Perspectives in Social and Behavioral scIences. Global Perspectives on Teacher Motivation*, eds Watt H. M. G. Richardson P. W. Smith K. (Cambridge: Cambridge University Press), 322–348. 10.1017/9781316225202.011

[B50] Levinson W. Gordon G. Skeff K. (1990). Retrospective versus actual pre-course self-assessments. *Eval. Health Prof.* 13 445–452. 10.1177/016327879001300406

[B51] Longfield D. Tooley J. (2017). School choice and parental preferences in a poor area of Monrovia. *Int. J. Educ. Dev.* 53 117–127. 10.1016/j.ijedudev.2016.08.006

[B52] Lotter C. Smiley W. Thompson S. Dickenson T. (2016). The impact of a professional development model on middle school science teachers’ efficacy and implementation of inquiry. *Int. J. Sci. Educ.* 38 2712–2741. 10.1080/09500693.2016.1259535

[B53] Lotter C. Thompson S. Dickenson T. Smiley W. F. Glue G. Rea M. (2018). The impact of a practice-teaching professional development model on Teachers’ Inquiry instruction and inquiry efficacy beliefs. *Int. J. Sci. Math Educ.* 16 255–273. 10.1007/s10763-016-9779-x

[B54] MacNabb C. Brier G. Teegarten J. Schmitt L. Drager N. Thomas L. (2006a). *Lessons. BrainU Website**/Resources.* Availablre online at: http://brainu.org/lesson-table (accessed January 1, 2021).

[B55] MacNabb C. Schmitt L. Michlin M. Harris I. Thomas L. Chittendon D. (2006b). Neuroscience in middle schools: a professional development and resource program that models inquiry-based strategies and engages teachers in classroom implementation. CBE. *Life Sci. Educ.* 5 144–157. 10.1187/cbe.05-08-0109 17012205 PMC1618517

[B56] Matteucci M. C. Guglielmi D. Lauermann F. (2017). Teachers’ sense of responsibility for educational outcomes and its associations with teachers’ instructional approaches and professional wellbeing. *Soc. Psychol. Educ.* 20 275–298. 10.1007/s11218-017-9369-y

[B57] Meltzoff A. N. Kuhl P. K. Movellan J. Sejnowski T. J. (2009). Foundations for a new science of learning. *Science* 325 284–288. 10.1126/science.1175626 19608908 PMC2776823

[B58] MTA Cooperative Group (1999). A 14-month randomized clinical trial of treatment strategies for attention-deficit/hyperactivity disorder. The MTA cooperative group. Multimodal treatment study of children with ADHD. *Arch. Gen. Psychiatry* 56 1073–1086. 10.1001/archpsyc.56.12.1073 10591283

[B59] Nitsche S. Dickhauser O. Fasching M. S. Dresel M. (2013). Teachers’ professional goal orientations: importance for further training and sick leave. *Learn. Individ. Diff.* 23 272–278. 10.1016/j.lindif.2012.07.017

[B60] Nolen S. B. Ward C. W. Horn I. S. (2014). “Changing practices. A situative account of teachers’ motivations to learn,” in *Teacher Motivation : Theory and Practice*, eds Richardson P. W. Karabenick S. A. Watt H. M. G. (Abingdon: Taylor & Francis Group), 167–181.

[B61] OECD (2008). *TALIS 2008 Technical Report.* Paris: OECD.

[B62] Open Society Foundations (2015). *Partnership Paradox. The Post-Conflict Reconstruction of Liberia’s Education System.* New York, NY: C. Talbot & A. Taylor.

[B63] Osher D. Coggshal J. Colombi G. Woodruff D. Francois S. Osher T. (2012). Building school and teacher capacity to eliminate the school-to-prison pipeline. *Teach. Educ. Spec. Educ.* 35 284–295. 10.1177/0888406412453930

[B64] Osher D. Kidron Y. Brackett M. A. Dymnicki A. Jones S. Weissberg R. P. (2016). Advancing the science and practice of social and emotional learning: looking back and moving forward. *Rev. Res. Educ.* 40 644–681. 10.3102/0091732x16673595

[B65] Osman D. J. Warner J. R. (2020). Measuring teacher motivation: The missing link between professional development and practice. *Teach. Teach. Educ.* 92:103064. 10.1016/j.tate.2020.103064

[B66] Owens M. T. Tanner K. D. (2017). Teaching as brain changing: exploring connections between neuroscience and innovative teaching. *CBE Life Sci. Educ.* 16:fe2. 10.1187/cbe.17-01-0005 28450442 PMC5459260

[B67] Pacione L. Cooper J. (2014). *Manual of School Mental Health. Adapted version for Liberia.* Cairo: Eastern Mediterranean Regional Office: W.H. Organization.

[B68] Palmer D. H. (2006). Sources of self-efficacy in a science methods course for primary teacher education students. *Res. Sci. Educ.* 36 337–353. 10.1007/s11165-005-9007-0

[B69] Perez D. Van Der Stuyft P. Del Carmen Zabala M. Castro M. Lefevre P. (2016). A modified theoretical framework to assess implementation fidelity of adaptive public health interventions. *Implement. Sci.* 11:91. 10.1186/s13012-016-0457-8 27391959 PMC4939032

[B70] Posner M. I. Rothbart M. K. (2007). *Educating the Human Brain.* Washington DC: American Psychological Association.

[B71] Pritchett L. Sandefur J. (2013). Context matters for size: why external validity claims and development practice do not mix. *J. Glob. Dev.* 4 161–197.

[B72] Revai N. Guerriero S. (2017). “Knowledge dynamics in the teaching profession,” in *Pedagogical Knowledge and the Changing Nature of the Teaching Profession*, ed. Guerriero S. (Paris: OECD Publishing), 99–118.

[B73] Richardson P. W. Watt H. M. G. (2014). “Why people choose teaching as a career. An expectancy-value approach to understanding teacher motivation,” in *Teacher Motivation: Implications for Theory and Practice*, eds Richardson P. W. Karabenick S. A. Watt H. M. G. (New York, NY: Routledge), 3–19. 10.4324/9780203119273-1

[B74] Roehrig G. H. Michlin M. Schmitt L. Macnabb C. J. Dubinsky J. M. (2012). Teaching neuroscience to science teachers: facilitating the translation of inquiry-based teaching instruction to the classroom. *CBE Life Sci. Educ.* 11 413–424. 10.1187/cbe.12-04-0045 23222837 PMC3516797

[B75] Romero M. Sandefur J. (2019). *Beyond Short-term Learning Gains: The Impact of Outsourcing Schools in Liberia after Three Years.* Washington, DC: Center For Global Development.

[B76] Roth K. J. Wilson C. D. Taylor J. A. Stuhlsatz M. A. M. Hvidsten C. (2019). Comparing the effects of analysis-of-practice and content-based professional development on teacher and student outcomes in science. *Am. Educ. Res. J.* 56 1217–1253. 10.3102/0002831218814759

[B77] Saldana J. (2016). *The Coding Manual for Qualitative Researchers.* London: SAGE Publications Ltd.

[B78] Saunders R. (2013). The role of teacher emotions in change: experiences, patterns and implications for professional development. *J. Educ. Chang.* 14 303–333. 10.1007/s10833-012-9195-0

[B79] Schonert-Reichl K. A. Kitil J. Hanson-Peterson J. (2017). *To Reach the Students, Teach the Teachers. A National Scan Of Teacher Preparation and Social & Emotional Learning. A Report Prepared for the Collaborative for Academic, Social, and Emotional Learning (CASEL).* Vancouver BC: University of British Colombia.

[B80] Schwartz M. S. Hinesley V. Chang Z. Dubinsky J. M. (2019). Neuroscience knowledge enriches pedagogical choices. *Teach. Teach. Educ.* 83 87–98. 10.1016/j.tate.2019.04.002

[B81] Selemani-Meke E. (2013). Teacher motivation and implementation of continuing professional development programmes in Malawi. *Anthropologist* 15 107–115. 10.1080/09720073.2013.11891297

[B82] SFN (2019). *BrainFacts.org [Online].* Washington DC: Society for Neuroscience.

[B83] Shulman L. S. (1986). Those who understand: knowledge growth in teaching. *Educ. Res.* 15 4–14. 10.3102/0013189x015002004

[B84] Shulman L. S. (1987). Knowledge and teaching: foundations of the new reform. *Harvard Educ. Rev.* 57 1–22. 10.4324/9781351233866-1

[B85] Shulman L. S. Shulman J. H. (2004). How and what teachers learn: a shifting perspective. *J. Curric. Stud.* 36 257–271. 10.1080/0022027032000148298

[B86] Sonmark K. Revai N. Gottschalk F. Deligiannidi K. Burns T. (2017). *Understanding Teachers’ Pedagogical Knowledge: Report on an International Pilot Study.* Paris: OECD, 10.1787/43332ebd-en

[B87] Thoonen E. E. J. Sleegers P. J. C. Oort R. J. Peetsma T. T. D. Geijsel F. P. (2011). How to improve teaching practices: the role of teacher motivation, organizational factors, and leadership practices. *Educ. Adm. Q.* 47 496–536. 10.1177/0013161x11400185

[B88] Tschannen-Moran M. Hoy A. W. (2001). Teacher efficacy: capturing an elusive construct. *Teach. Teach. Educ.* 17 783–805. 10.1016/s0742-051x(01)00036-1

[B89] Vieluf S. Kunter M. Van De Vijvier F. J. R. (2013). Teacher self-efficacy in cross-national perspective. *Teach. Teach. Educ.* 35 92–103. 10.1016/j.tate.2013.05.006

[B90] Voss T. Kunter M. Baumert J. (2011). Assessing teacher candidates’ general pedagogical/psychological knowledge: test construction and validation. *J. Educ. Psychol.* 103 952–969. 10.1037/a0025125

[B91] Weist M. D. Bruns E. J. Whitaker K. Wei Y. Kutcher S. Larsen T. (2017). School mental health promotion and intervention: experiences from four nations. *Sch. Psychol. Int.* 38 343–362. 10.1177/0143034317695379

[B92] Westbrook J. Durrani N. Brown R. Orr D. Pryor J. Boddy J. (2013). *Pedagogy, Curriculum, Teaching Practices and Teacher Education in Developing Countries.* Final Report. Sussex: D.F.I. Development.

[B93] Williams J. H. (2011). Education and reconstruction in post-conflict Liberia. *J. Int. Coop. Stud.* 18 55–79.

[B94] Wolfenden F. Buckler A. Keraro F. (2012). OER adaptation and reuse across cultural contexts in Sub Saharan Africa: lessons from TESSA (Teacher Education in Sub Saharan Africa). *J. Interact. Media Educ.* 2012:3. 10.5334/2012-5303

[B95] Yeager D. S. Hanselman P. Walton G. M. Murray J. S. Crosnoe R. Muller C. (2019). A national experiment reveals where a growth mindset improves achievement. *Nature* 573 364–369. 10.1038/s41586-019-1466-y 31391586 PMC6786290

[B96] Zee M. Koomen H. M. Y. (2016). Teacher self-efficacy and its effects on classroom processes, student academic adjustment, and teacher well-being: a synthesis of 40 years of research. *Rev. Educ. Res.* 86 981–1015. 10.3102/0034654315626801

